# Analysis of Host–Bacteria Protein Interactions Reveals Conserved Domains and Motifs That Mediate Fundamental Infection Pathways

**DOI:** 10.3390/ijms231911489

**Published:** 2022-09-29

**Authors:** Jordi Gómez Borrego, Marc Torrent Burgas

**Affiliations:** Systems Biology of Infection Laboratory, Department of Biochemistry and Molecular Biology, Biosciences Faculty, Universitat Autònoma de Barcelona, 08193 Bellaterra, Spain

**Keywords:** pathogen, host, protein interaction, domain, motif, Alphafold

## Abstract

Adhesion and colonization of host cells by pathogenic bacteria depend on protein–protein interactions (PPIs). These interactions are interesting from the pharmacological point of view since new molecules that inhibit host-pathogen PPIs would act as new antimicrobials. Most of these interactions are discovered using high-throughput methods that may display a high false positive rate. The absence of curation of these databases can make the available data unreliable. To address this issue, a comprehensive filtering process was developed to obtain a reliable list of domains and motifs that participate in PPIs between bacteria and human cells. From a structural point of view, our analysis revealed that human proteins involved in the interactions are rich in alpha helix and disordered regions and poorer in beta structure. Disordered regions in human proteins harbor short sequence motifs that are specifically recognized by certain domains in pathogenic proteins. The most relevant domain–domain interactions were validated by AlphaFold, showing that a proper analysis of host-pathogen PPI databases can reveal structural conserved patterns. Domain–motif interactions, on the contrary, were more difficult to validate, since unstructured regions were involved, where AlphaFold could not make a good prediction. Moreover, these interactions are also likely accommodated by post-translational modifications, especially phosphorylation, which can potentially occur in 25–50% of host proteins. Hence, while common structural patterns are involved in host–pathogen PPIs and can be retrieved from available databases, more information is required to properly infer the full interactome. By resolving these issues, and in combination with new prediction tools like Alphafold, new classes of antimicrobials could be discovered from a more detailed understanding of these interactions.

## 1. Introduction

Protein–protein interactions (PPIs) play a fundamental role in most biological processes. Infectious diseases are no exception, as pathogens rely on PPIs to attach to and infect host cells [1,2,3]. In fact, the fitness defect resulting from the deletion of a gene in the pathogen depends on the number of interactions that the corresponding protein can exert with the host [4]. Hence, the analysis of central proteins in the host–pathogen interactome is a promising strategy to identify new targets for antibiotic drug design [5,6,7,8]. In this context, several databases have compiled experimental evidence of host–pathogen PPIs, involving viruses, bacteria, and fungi [9,10,11,12,13]. Most of these interactions involve pathogenic proteins with human targets. However, such databases also contain many spurious interactions, due to the high false positive rate from high-throughput assays [14,15,16]. This is a critical issue, as almost all PPI prediction algorithms are trained on experimentally validated data.

Even more important, is a proper understanding of the conserved structural patterns in host–pathogen PPIs. A deeper study of these interactions, i.e., the prediction of the three-dimensional structures of the interacting proteins, will allow us to characterize pathogenesis at the molecular level and identify the most promising pharmacological targets.

Protein domains are regions that can fold, function, and evolve independently and are used as building blocks that can be combined to build proteins with different functions. Various databases such as iPfam [17], 3did [18], or the Database of Protein Domain Interactions (DOMINE) [19] contain information on protein domains and domain interactions, mostly extracted from experimental evidence such as structures deposited in the Protein Data Bank (PDB) [20]. Since most proteins contain multiple domains, the interaction between two proteins likely involves the contact of two or more domains. In fact, PPIs are thought to be primarily based on domain interactions [21], but there are thousands of possible combinations and not all of them are present in known PPIs. Hence, studying domain associations is an interesting approach to gain insight into the structural details behind protein interactions.

Although domains are important in characterizing PPIs, many of the interactions occur between domains and unstructured, more discrete elements, known as motifs. Motifs are short (typically 3–10 residues) conserved and regulatory protein components that provide low-affinity interaction interfaces, and are usually found in intrinsically disordered regions (IDRs) [22]. These components are often specialized for protein-binding functions and have a central role in cell signaling and protein localization [23]. Due to their short length, motifs are often degenerate, making motif prediction unreliable in most cases. Only a few classes of motifs have been defined thanks to experimental evidence. These motifs are included in databases such as Eukaryotic Linear Mofif (ELM) Database [24], which contains hundreds of annotated motifs stored as regular expressions (RegExp) to help researchers predict biologically relevant motifs. Such motifs interact with protein domains to mediate key cellular processes, including phosphorylation, glycosylation, and ubiquitination.

Here, a new filtering pipeline was created to screen PPIs between host and pathogenic bacteria (hereafter host-pathogen PPIs) by considering enriched protein domains and motifs to identify central interactions for pathogenesis. The refined list of domain–domain and domain–motif interactions enriched in host–pathogen PPIs shows that the number of statistically relevant interactions in these datasets is limited, suggesting that more data is required to define the host–pathogen interactome. Notwithstanding, several structural patterns can be obtained from current databases that may help to pave the way for the development of new antimicrobials.

## 2. Results and Discussion

Our goal was to retrieve the relevant structural information from host–pathogen PPI databases, minimizing noise interference. The Pathogen-Host Interaction Search Tool (PHISTO) database currently represents the most comprehensive repository of human–bacterial interactions [10]. For this reason, the domains and motifs present in the PHISTO database were retrieved and analyzed for relevant structural elements and to understand how these elements interact with each other.

**Figure 1:**
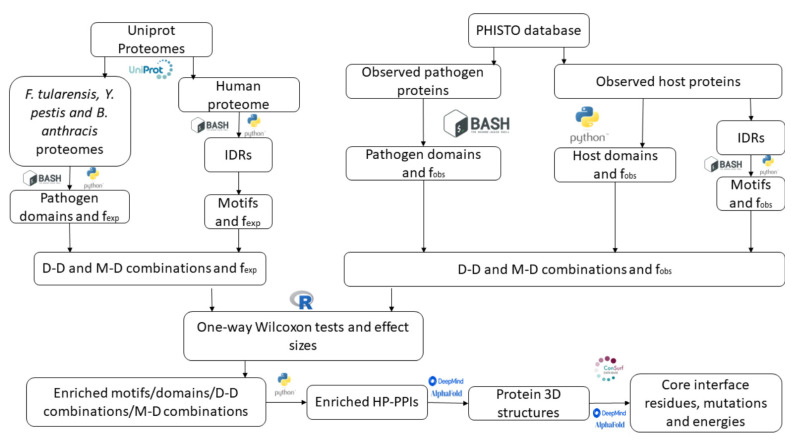
Workflow followed to retrieve HP-PPIs from the PHISTO database considering enriched motifs, domains, domain–domain and domain–motif associations, and to analyze the interface regions.

To find domains and motifs involved in host–pathogen PPIs, 9.333 PPIs between bacteria and human proteins were retrieved from the PHISTO database, 9.027 of which were unique interactions. These interactions are formed by 2.716 bacterial proteins and 3.737 human proteins. Here, InterProScan [25] was used to identify domains from the protein sequences. Among these interactions, 2.539 unique domains were identified in human proteins and 1.898 in pathogen proteins. Then, the EBI-Alphafold database [26] of protein structures was used to identify disordered regions in human proteins, and the RegEx definition of motifs in the ELM database [24] was used to detect motifs. Intrinsically disordered regions (IDRs) were identified in 2.907 out of 3.519 human proteins and detected ELM motifs in 2.860 of these proteins. These results were used to build a pipeline and detect enriched domains, motifs, domain–domain interactions, and domain–motif interactions (Figure 1). In the following sections, enriched instances in the PHISTO database were used to investigate their relevance to the infection process. Additional details are provided in the Methods section.

### 2.1. Enriched Domains in Host–Pathogen PPIs

**Figure 2:**
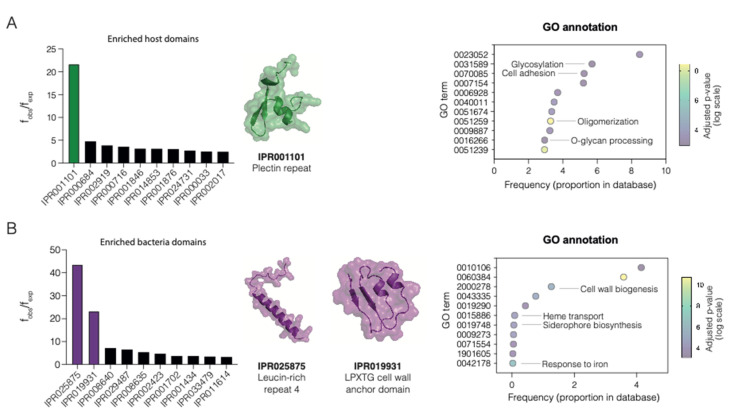
Analysis of enriched domains in host-pathogen PPIs. (**A**) Bar plot representation of the 10 most enriched domains for human proteins (left). The tridimensional structure of the most enriched human domain, IPR001101, is displayed. Representative GO enriched terms for host domains are displayed on the right. (**B**) Bar plot representation of the 10 most enriched observed domains for bacterial proteins (left). The tridimensional structure of the two most enriched bacterial domains, IPR025875 and IPR019931, are also displayed. Representative GO enriched terms for bacterial domains are displayed on the right. In all cases, the GO term frequency is displayed on the x-axis and the GO term on the y-axis. Colors represent adjusted *p*-values for each GO term as calculated by dcGO [31].

A total of 48 host and 69 pathogen domains (Supplementary Materials) were found to be enriched in the PHISTO database, being the plectin repeat domain (IPR001101) the most enriched one, with an observed frequency 21.69 times higher than expected (Figure 2A). The rest of the overrepresented human domains were observed from 2.49 to 4.77 times their expected frequencies. Plectin has a central role in the cell cytoskeleton and is involved in crosslinking of intermediate filaments [27]. It provides linkage between the keratin filaments inside the cell and the laminins in the extracellular matrix. Cytoskeleton remodeling is a central process in bacterial infections that allows internalization and dissemination of bacteria. For example, in *Acinetobacter baumanii*, several virulence factors (lipoproteins, OmpA, and Lon protease) were found to interact with cytoskeleton proteins, including plectin [28]. Enriched Gene Ontology (GO) terms for human proteins include cell adhesion and glycosylation, both relevant to pathogen adherence to the cell matrix (Figure 2A). Regarding the pathogen domains, a similar pattern was observed (Figure 2B). Two domains were highly enriched: IPR025875, a leucine-rich repeat, and IPR019931, the LPXTG cell wall anchor domain. Other domains showed enrichment frequencies from 3 to 7 times higher than expected. Leucine-rich repeats are found in bacterial surface proteins and are associated with PPIs, such as internalins in *Listeria monocytogenes*, used to invade mammalian cells via cadherins transmembrane proteins [29]. LPXTG cell wall anchor domains are surface proteins commonly found in Gram-positive bacteria, including pilus, fimbria, and adhesins [30]. A GO enrichment analysis of the domains retrieved shows relevant functions related to bacterial infection, such as cell wall biogenesis and siderophore biosynthesis (Figure 2B). 

**Figure 3:**
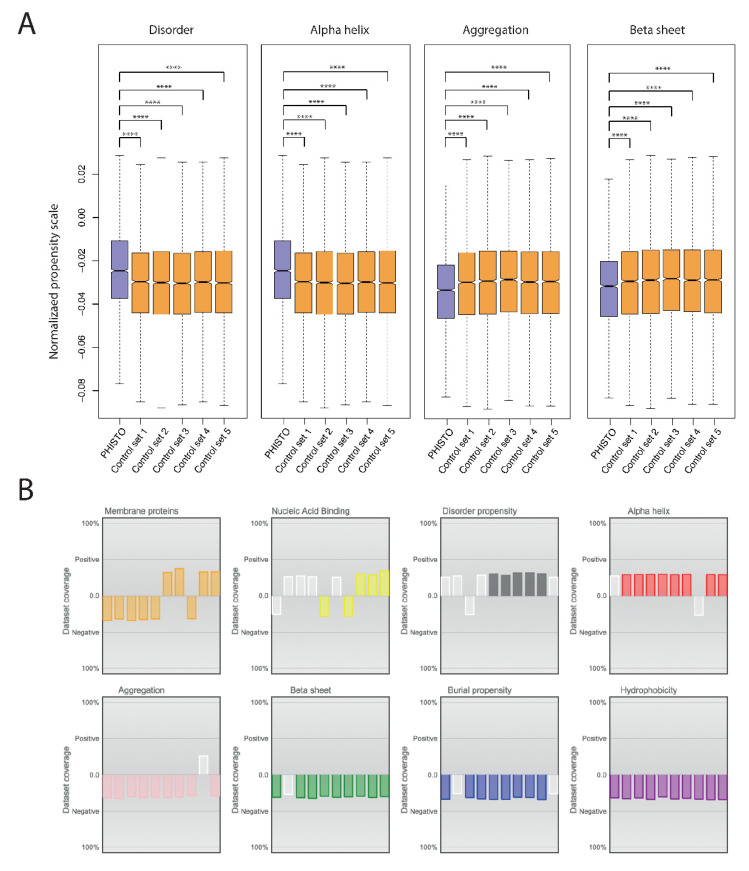
Physicochemical and structural properties of human proteins participating in host–pathogen interactions. (**A**) Boxplot representation of major properties for human proteins in PPIs compared to five groups of human proteins selected by random picking from the human proteome. (**B**) Overall representation of all scales used in CleverMachine for evaluating features in proteins. For a list of all properties evaluated, see [32]. Statistical comparisons were made using the Mann-Whitney U-test. **** *p* ≤ 0.0001.

Additionally, the human proteins involved in host–pathogen PPIs were inspected for specific structural and physicochemical properties. For this, protein sequences were inspected with Clever Machine [32], an algorithm used to discriminate between two sets of proteins using physicochemical properties encoded in their sequences. The human proteins involved in host–pathogen PPIs were compared with five random sets of human proteins with the same sample size and similar size distribution (Figure 3A). The results show that human proteins involved in host–pathogen PPIs are specifically enriched in disordered regions and rich in alpha helix, while they are depleted in beta-sheet and aggregation-prone regions. The results suggest that human proteins targeted by pathogens have singular structural features. The results obtained are not biased for the propensity scales used, as different scales give similar results (Figure 3B). These proteins are also depleted in membrane proteins and enriched in nucleic acid-binding proteins, which could suggest an enrichment in specific functions, e.g., transcription factors or ribonucleoprotein interacting proteins.

### 2.2. Domain–Domain Associations in Host–Pathogen PPIs

**Figure 4:**
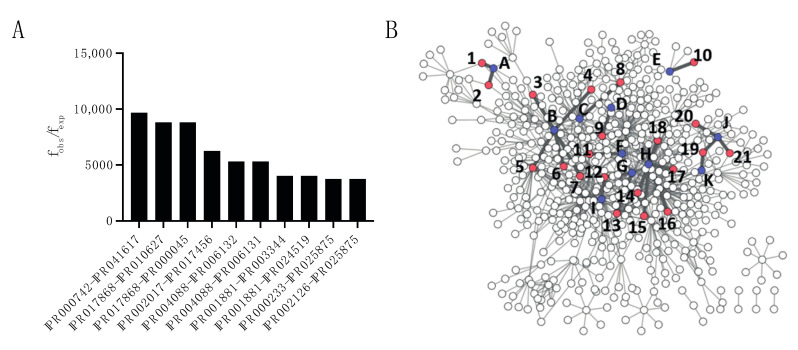
Analysis of domain–domain associations. (**A**) Bar graph representation of the 10 most enriched DD associations. (**B**) Network representation of the DD associations in the PHISTO dataset. Interactions highlighted in dark gray represent the enriched DD associations present in at least three interactions. Host proteins are identified by letters and bacterial proteins by numbers. More details on these proteins can be found in Table 1.

**Figure 5:**
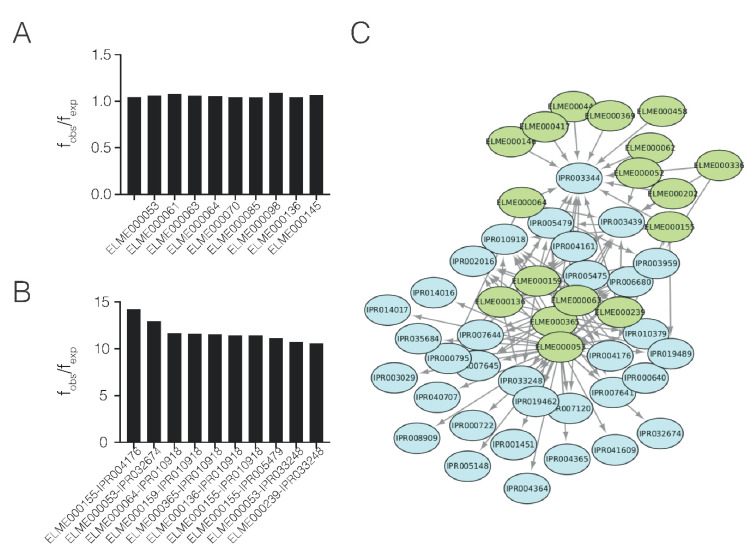
Analysis of enriched motifs and domain–motif associations. (**A**) Bar plot representation of the 10 most enriched motifs and (**B**) domain-motif combinations. (**C**) Network representation of domain–motif associations. Domains are colored in blue and motifs in green.

As many PPIs are driven by domain interactions, certain domain associations might be privileged in host–pathogen PPIs. To do this, the frequency of all occurring domain–domain (DD) associations were calculated and compared with the expected frequency if associations would occur at random. The 10 most enriched DD associations are depicted in Figure 4A. Most of the observed 149 enriched domain associations occur in one or two different interactions while 21 of them were counted in three or more different interactions. A network of all DD associations is displayed in Figure 5B, with the 21 most frequent interactions highlighted and listed in Table 1 and Supplementary Materials.

### 2.3. Domain–Motif Associations in Host–Pathogen PPIs

Many processes in the cell, such as phosphorylation or ubiquitination, are mediated by transient PPIs that occur via domain–motif (DM) interactions. As human proteins involved in host–pathogen PPIs contain large, disordered regions, these proteins were further scanned for enriched motifs. Among the human proteins involved in host–pathogen PPIs, 29 enriched motifs were found (Figure 5A). The ratio between observed and expected frequencies (f_obs_/f_exp_) of the enriched motifs were less marked than domains. The most enriched motifs were the WDR5-binding motif (ELM000364) and the COP1 E3 ligase-binding degron motif, involved in histone methylation and ubiquitination, respectively. 

**Figure 6:**
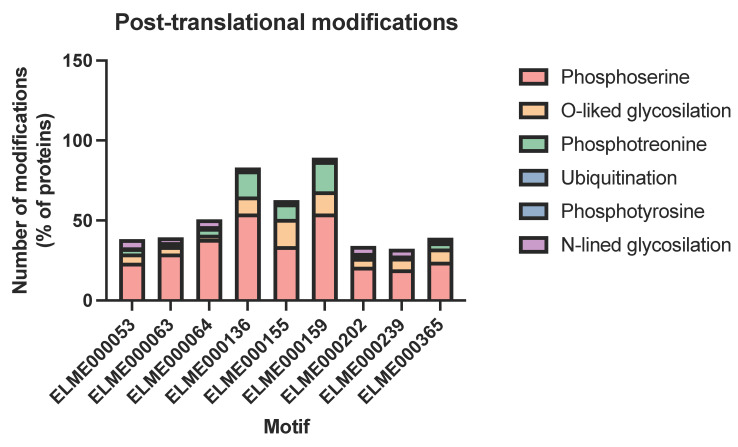
Post-translational modifications in enriched motifs. The sequences were inspected using MusiteDeep [36] and modifications were reported as the percentage of proteins containing a given modification for each motif.

A total of 75 enriched domain–motif (DM) associations were found in the database. (Figure 5B, Supplementary Materials). Among the most overrepresented combinations (Figure 6) were the ClpR domain (IPR004176), an LRR-containing E3 ligase (IPR032674), and a PurM-like domain. The Clp domain is related to the bacterial Clp/Hsp100 family of chaperones and has a major role in biofilm formation and virulence, facilitating the initial attachment of bacteria to surfaces [33]. Additionally, LRR-containing ligases are secreted by type III effectors [34]. Finally, PurM domains are related to dehydratase enzymes, with some evidence linked to virulence [35]. The network representation of the motifs and domains (Figure 5C) shows that most of the connections involve only a few motifs, meaning that each motif can interact with several domains.

Motifs are susceptible to modulation by post-translational modifications, which can notably modify the interactions. Hence, we investigated whether the motifs present in DM associations could be modified. We used the MusiteDeep [36] tool that uses deep learning to predict post-translational modifications in proteins. As expected, the motifs can be heavily modified (Figure 6), particularly motifs ELME000136 (Group IV WW substrates), ELME000155 (SH3-binding motif), and ELME000159 (MAPK phosphorylation site). These motifs are also susceptible to O-linked glycosylation, particularly ELME000155.

### 2.4. Structural Analysis of DD and DM Associations

**Figure 7:**
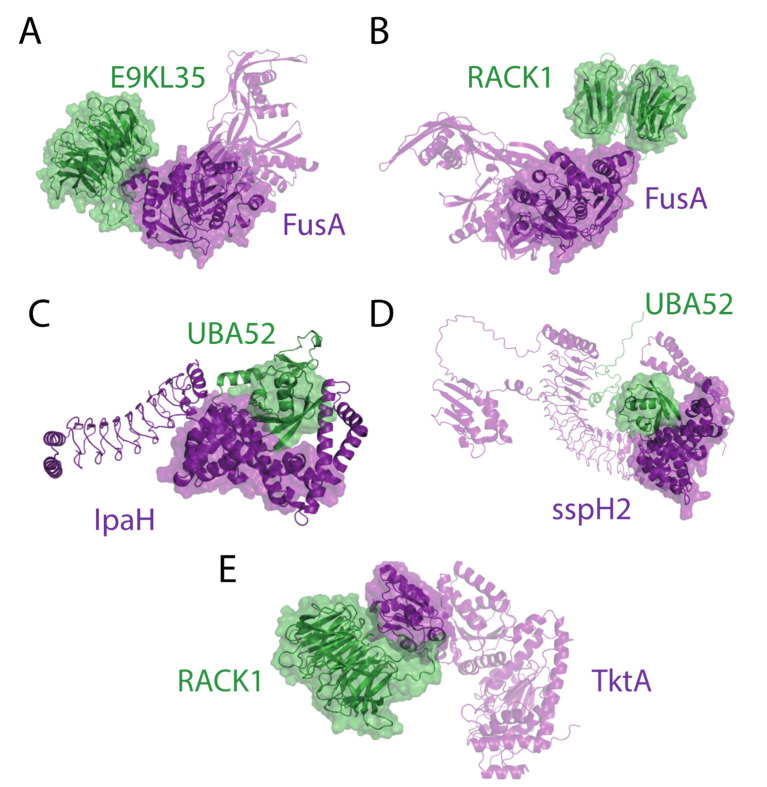
Domain–domain interactions predicted by Alphafold Multimer. (**A**) E9KL35-FusA; (**B**) RACK1-FusA; (**C**) UBA52-IpaH; (**D**) UBA52-sspH2; (**E**) RACK1-TktA.

From the 21 enriched DD and 75 DM interactions, representative complexes were modeled using AlphaFold Multimer [37,38]. We restricted the cases where the total length of the proteins of the interaction was lower than 1.500 residues. Proteins with low confidence regions (pLDDT score < 0.5) above 10% were filtered out. Using these constraints, 15 representative DD and 27 DM complexes were predicted. From 15 DD complexes, 5 of them had pTM scores above 0.5: fusA-E9KL35 (pTM score = 0.75), fusA-RACK1 (pTM score = 0.65), sspH2-UBA52 (pTM score = 0.62), ipaH9.8-UBA52 (pTM score = 0.58), tktA-RACK1 (pTM score = 0.57). In all cases, the associated domains were found in close proximity according to Alphafold predictions, suggesting that our pipeline correctly identifies DD associations with high confidence (Figure 7).

**Figure 8:**
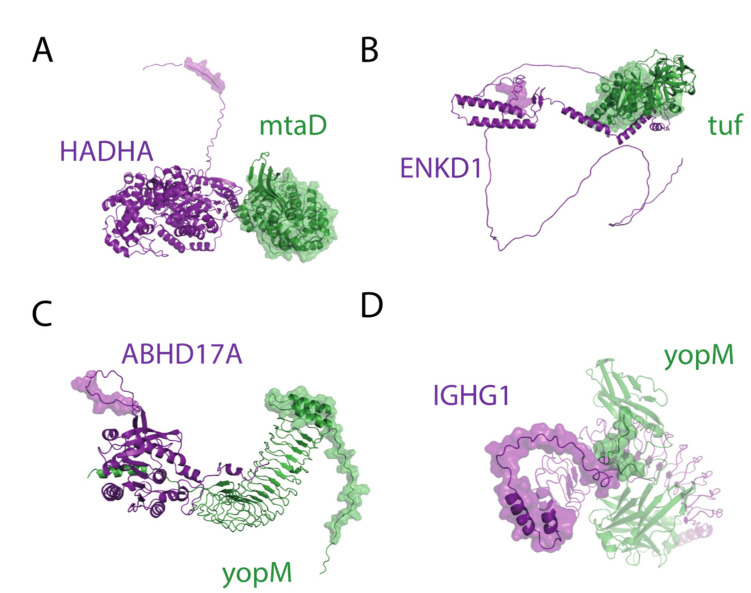
Domain–motif interactions predicted by Alphafold Multimer. (**A**) HADHA-mtaD; (**B**) ENKD1-tuf; (**C**) ABHD17A-yopM; (**D**) IGHG1-yopM.

The validation of DM interactions was more challenging. As motifs are located in disordered regions, our threshold for protein structure quality (less than 10% of residues with pLDDT score < 0.5) removed more than 75% of the total entries. From the 27 PPIs containing a DM association, the only accurate models correspond to yopM-ABHD17A (pTM score = 0.62), mtaD-HADHA (pTM score = 0.61), yopM-IGHG1 (pTM score = 0.56), and tuf-ENKD1 (pTM score = 0.55). In all cases, the motifs were located in highly flexible regions and not always in close proximity to the domain (Figure 8). Only yopM-IGHG1 had the domain and motif in close contact, probably because the motif in IGHG1 was included in a loop with restricted mobility. Hence, the presence of disordered regions is a clear limitation in predicting interactions by Alphafold Multimer. Despite not being in contact with the structures predicted, the high flexibility would still allow transient interactions to happen. Moreover, post-translational modifications can also have a role here, making the prediction of MD interactions even more challenging.

### 2.5. Virulence Factors with Ubiquitin–Protein Ligase Activity as a Case Example

We decided to analyze in more detail the complexes between IpaH and sspH2 with ubiquitin UBA52. These interactions involve the association of the ubiquitin domain in UBA52 with the catalytic domain of ubiquitin–protein ligase E3, both in IpaH and sspH2. The structure of IpaH and sspH2 is similar, with a ubiquitin–protein ligase domain and several leucine-rich repeats except for the additional domain at the N-terminus of sspH2. The ubiquitin ligase activity of these proteins is most likely not restricted to UBA52. In fact, the interaction between IpaH and UBC is also described in PHISTO and, when inspected with Alphafold, both proteins can potentially interact with other forms of ubiquitin, such as polyubiquitin B.

**Figure 9:**
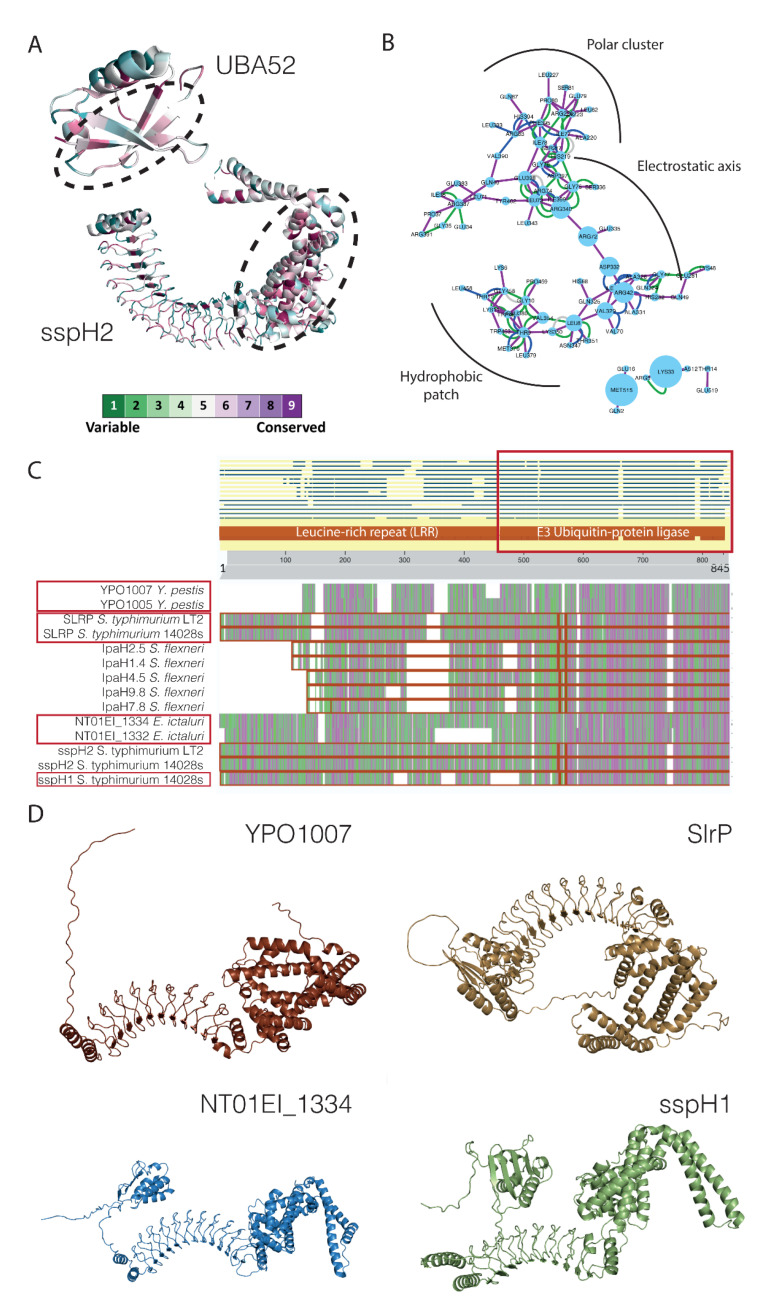
Analysis of complexes between IpaH and sspH2 with ubiquitin UBA52. (**A**) Structure of UBA52 and sspH2 showing the degree of sequence conservation. The conservation scale is displayed at the bottom of the figure. (**B**) Residue contact network between UBA52 and sspH2 showing several contact signatures. (**C**) Blast search results using the ubiquitin-protein ligase domain as the query sequence. Newly identified domains are highlighted by brown boxes. (**D**) Predicted structures of selected proteins using Alphafold [26].

To analyze the degree of conservation of the interacting regions, we use Consurf [39]. The analysis shows that the interface regions of both domains are more conserved than the rest of the protein (Figure 9A). This suggests that the interaction was correctly predicted since the interfaces of the complexes are usually more conserved than the rest of the surface [40]. The interacting residues can be located in three main regions, including an electrostatic axis, that contributes the most to the energy binding, and a polar and hydrophobic groove that modulates binding (Figure 9B). Indeed, the interfaces detected in human-binding partners correspond to interfaces predicted in other human–human interactions already described. These results suggest that the interactions detected by our pipelines are probably real and correspond to some kind of interface mimicry by pathogens [41].

## 3. Materials and Methods

### 3.1. Databases

Host–pathogen PPIs were retrieved from the PHISTO database (accessed 11 February 2022) [10] by accessing the web platform, using the “Browser” option and selecting “Bacteria” as the pathogen type and “All” on the family field, and finally, downloading the table containing the 9.333 HP-PPIs from which 9.027 represent unique interactions. The sequences of all the pathogen and human proteins were retrieved in Uniprot using the Uniprot ID mapper tool. The human proteome, as well as the proteomes of *Yersinia pestis* (UP000000815), *Bacillus anthracis* (UP000000594), and *Francisella tularensis* (UP000001174), were downloaded from Uniprot Proteomes in FASTA format.

### 3.2. Domain and Motif Scanning

InterProScan v5.56 [25] was run locally to locate protein domains for all the pathogen and human proteins. InterProScan implements the domain prediction from multiple analyses, including PANTHER, Pfam, and SUPERFAMILY. A simple bash script (interpro_to_pfam.sh) was used to iterate through all the FASTA files and select only the Pfam records, including the InterProScan identifier, the region where the domain is located (positions of the first and last residue), and a short description of the protein domain.

The list of ELM classes was downloaded in TSV format from the ELM database (accessed 21 February 2022) [24]. This list contains the ELM accessions and the regular expressions’ patterns for each class. As the motifs are usually found in IDRs, the prediction of these regions will define the motif search space. Alphafold pLDDT score was used as a disorder predictor [42]. The per-residue surface accessibility scores for all the human proteins based on the Alphafold structure predictions were retrieved from previous calculations by Bálint Mészáros and Norman Davey (https://github.com/normandavey/ProcessedAlphafold, accessed on 21 February 2022). A cut-off value higher than 0.55 was used to define whether a residue belongs to an IDR. A Python script (host_motifs.py) was built to select the disordered regions for each human protein given the per-residue surface accessibility scores, picking regions with a minimum length of 5 residues. The IDR regions for every human protein were stored in separate files. The same script was used to fetch all the motifs contained in IDRs for each human protein. The script searches for ELM patterns in disordered regions, as previously identified, and retrieves the positions of the matches.

### 3.3. Domain–Domain (DD) and Domain–Motif (DM) Interactions

DD and DM combinations were obtained by a brute force approach using Python (clean_phisto.py). The idea behind this approach was to iterate through every single PPI contained in the PHISTO database and generate all the possible domain–motif and domain–domain combinations. Then, all these possible combinations were processed to filter out the non-significant interactions by statistical analysis.

### 3.4. Domain and Motif Enrichment Analysis

To find which observed motifs, domains, DD, and DM combinations were enriched in the PHISTO database, their relative frequencies (observed frequencies) were calculated and compared to the relative frequencies that would result from random sampling (expected frequencies). All statistical calculations were performed in R. The observed frequencies of all the commented elements were computed by iterating through all the output files containing the motif/domain and counting how many times each element was observed in the PHISTO database with respect to the total number of elements. The results of this step were stored in text files containing the number of times a particular element appeared, the identifiers, and the relative frequencies.

To calculate the expected frequencies for human domains and motifs, 3.737 proteins from the human proteome were randomly picked to count how many times each domain or motif was observed with respect to the total number of elements. This step was repeated 1000 times using bootstrapping to get the expected frequency. For pathogen proteins, a single proteome would not be representative of the database, so a mixed proteome was created. Since 94% of the bacterial proteins registered in the PHISTO database belong to three bacterial species (44% *Y. pestis*, 33% *B. anthracis*, and 17% *F. tularensis*), in each bootstrap iteration, a random sampling of these proteomes, with identical percentages, was used to simulate as much as possible the conditions of the PHISTO database.

To compute the expected frequencies for domain–domain and domain–motif combinations, random combinations between the human proteome and the mixed proteome were used. Again, the expected frequency was calculated as the average frequency of 1.000 bootstrapping iterations.

Observed and expected frequencies were compared using the one-way Wilcoxon test. The output files containing the *p*-values from the Wilcoxon test comparison along with the effect size values were processed using a Python script (select_enriched.py) to select those elements whose *p*-values were below the significance level (α = 0.05) and whose effect sizes were above 0.5. The ratio between the observed frequency and the expected frequency (f_obs_/f_exp_) was computed for all the elements to classify them as enriched (f_obs_/f_exp_ > 1) or depleted (f_obs_/f_exp_ < 1). Only the enriched elements were further analyzed.

### 3.5. Gene Ontology Analysis

Gene Ontology (GO) Biological Process terms for the enriched human and pathogenic domains, as well as from the enriched DD combinations, were obtained using the Pfam identifiers in dcGO [31] (https://supfam.mrc-lmb.cam.ac.uk/SUPERFAMILY/cgi-bin/dcenrichment.cgi, accessed on 29 June 2022). The GO terms were later processed in REVIGO [43], a web server that summarizes lists of GO terms and finds the most representative terms relying on semantic similarities, generating a visual representation of non-redundant GO terms (http://revigo.irb.hr).

### 3.6. Structural and Conservation Analysis of Selected PPIs

The enriched DD and DM interactions were further explored using Alphafold Multimer [37,38]. Protein interactions containing enriched DD and DM interactions were retrieved and the structure was obtained. There were several limitations in this approach that precluded obtaining all interactions. The first one was the degree of disorder of the human protein. The structure of most human proteins was highly unfolded or contained a high percentage of very low confidence regions. Proteins showing a percentage of very low confidence regions (<50 pLDDT score) above 10% were discarded (263 out of 349 selected human proteins were discarded). The bacterial counterparts of the interactions successfully predicted by AlphaFold were analyzed using the ConSurf server [39] (https://consurf.tau.ac.il, all parameters set by default).

## 4. Conclusions

In this study, we have demonstrated the usefulness of using statistical tools to detect structural associations in PPIs databases that may contain many false positives. Using these tools, we were able to generate a list of domain–domain and domain–motif associations with a high degree of confidence. This information may be useful to validate or predict new bacterial proteins involved in infection, as shown in the case of virulence factors associated with ubiquitin–protein ligase activity. Through sequence alignment on the identified motif, possible virulence factors can be detected in other species, such as *Yersinia pestis* or *Edwardiella ictaluri* (Figure 9C). The presence of similar domains allows us to suggest the interaction between these proteins and human ubiquitin. This information, combined with Alphafold’s predictability, opens up a wide range of possibilities. In this case, the structure predicted for the potential virulence factors of *Y. pestis* and *E. ictaluri* have a high degree of similarity with the sspH2 protein, which increases the reliability of these predictions (Figure 9D). However, the main limitation in the study of host–pathogen PPIs still relies on the scarce amount of high-quality information available. At present, we can only identify the most common structural patterns that arise from this limited information. As more data becomes available, we will be able to better define the subtleties in the host–pathogen interactome.

Alphafold allows us to also evaluate if an interaction could be accurate. In our case, all the interactions predicted by Alphafold with a high degree of confidence confirmed that the identified domain–domain associations were consistent with the prediction. It is important to note here that there are limitations to this approach. The presence of important unstructured regions in human proteins involved in host–pathogen PPIs makes the predictions less confident. The high degree of flexibility of these structures means that these interactions can be, in many cases, transient. Unless they are part of loops connecting defined secondary structures, with limited flexibility, the prediction will almost certainly be poor.

The increase in the ability to predict new host–pathogen PPIs at the structural level opens the door to the in silico design of new drugs that inhibit these interactions. Considering that infections caused by resistant bacteria are a major public health problem, these new molecules could be a formidable contribution to the arsenal of already available antimicrobials.

## Figures and Tables

**Table 1:** InterPro identifiers and short descriptions of the host and pathogenic enriched domains, depicted in Figure 5B.

Network Identifier (Host)	InterPro Identifier (Host)	Description	Network Identifier (Pathogen)	InterPro Identifier (Pathogen)	Description
A	IPR001715	Calponin homology domain	1	IPR014016	UvrD-like helicase, ATP-binding domain
			2	IPR014017	UvrD-like DNA helicase, C-terminal
B	IPR000504	RNA recognition motif domain	3	IPR003343	Bacterial Ig-like domain, group 2
			4	IPR032781	ABC-transporter extension domain
			5	IPR003344	Big-1 domain
			6	IPR002314	Aminoacyl-tRNA synthetase, class II (G/P/S/T)
			7	IPR018392	LysM domain
C	IPR003961	Fibronectin type III	8	IPR019931	LPXTG cell wall anchor domain
D	IPR001245	S-T/Y-protein kinase	9	IPR010918	PurM-like, C-terminal domain
E	IPR000626	Ubiquitin-like domain	10	IPR029487	Novel E3 ligase domain
F	IPR001781	Zinc finger, LIM-type	11	IPR006680	Amidohydrolase-related
G	IPR001007	VWFC domain	12	IPR001036	Acriflavin resistance protein
			13	IPR007642	RNA polymerase Rpb2, domain 2
H	IPR001680	WD40 repeat	14	IPR004161	Translation elongation factor EFTu-like, domain 2
			15	IPR005475	Transketolase-like, pyrimidine-binding domain
			16	IPR033248	Transketolase, C-terminal
			17	IPR005474	Transketolase, N-terminal
			18	IPR000795	Translational (tr)-type GTP-binding domain
I	IPR001881	EGF-like calcium-binding domain	12	IPR001036	Acriflavin resistance protein
J	IPR000157	Toll/interleukin-1 receptor homology (TIR) domain	19	IPR001029	Flagellin, N-terminal domain
			20	IPR002423	Chaperonin Cpn60/GroEL/TCP-1 family
			21	IPR001702	Porin, Gram-negative type
K	IPR000488	Death domain	19	IPR001029	Flagellin, N-terminal domain

## Data Availability

All original datasets are publicly available at the detailed websites. All datasets generated are available as Supplementary Materials. All code used to generate the data are available at https://github.com/SysBioUAB/motif-domain_scripts.

## Supplementary Materials

The following supporting information can be downloaded at: https://www.mdpi.com/article/10.3390/ijms231911489/s1.
